# Fontan-associated liver disease: Diagnosis, surveillance, and management

**DOI:** 10.3389/fped.2023.1100514

**Published:** 2023-03-03

**Authors:** Charlotte de Lange, Thomas Möller, Hanna Hebelka

**Affiliations:** ^1^ Department of Pediatric Radiology, Queen Silvia Children’s Hospital, Sahlgrenska University Hospital, Gothenburg, Sweden; ^2^ Institution of Clinical Sciences, Sahlgrenska Academy, University of Gothenburg, Gothenburg, Sweden; ^3^ Department of Pediatric Cardiology, Oslo University Hospital, Oslo, Norway

**Keywords:** Fontan procedure, chronic liver disease, imaging, surveillance, congestion

## Abstract

The Fontan operation is a lifesaving procedure for patients with functional single-ventricle congenital heart disease, where hypoplastic left heart syndrome is the most frequent anomaly. Hemodynamic changes following Fontan circulation creation are now increasingly recognized to cause multiorgan affection, where the development of a chronic liver disease, Fontan-associated liver disease (FALD), is one of the most important morbidities. Virtually, all patients with a Fontan circulation develop liver congestion, resulting in fibrosis and cirrhosis, and most patients experience childhood onset. FALD is a distinctive type of congestive hepatopathy, and its pathogenesis is thought to be a multifactorial process driven by increased nonpulsatile central venous pressure and decreased cardiac output, both of which are inherent in the Fontan circulation. In the advanced stage of liver injury, complications of portal hypertension often occur, and there is a risk of developing secondary liver cancer, reported at young age. However, FALD develops with few clinical symptoms, a surprisingly variable degree of severity in liver disease, and with little relation to poor cardiac function. The disease mechanisms and modifying factors of its development are still not fully understood. As one of the more important noncardiac complications of the Fontan circulation, FALD needs to be diagnosed in a timely manner with a structured monitoring scheme of disease development, early detection of malignancy, and determination of the optimal time point for transplantation. There is also a clear need for consensus on the best surveillance strategy for FALD. In this regard, imaging plays an important role together with clinical scoring systems, biochemical workups, and histology. Patients operated on with a Fontan circulation are generally followed up in cardiology units. Ultimately, the resulting multiorgan affection requires a multidisciplinary team of healthcare personnel to address the different organ complications. This article discusses the current concepts, diagnosis, and management of FALD, with special emphasis on the role of different imaging techniques in the diagnosis and monitoring of disease progression, as well as current recommendations for liver disease surveillance.

## 1. Introduction

Children with univentricular heart disease surgically palliated with a Fontan circulation have experienced remarkably increased survival into adulthood during the last few decades, up to 61%–85% worldwide (1–4). Even better results have been reported for patients operated on after 2001, who have a 95% 10-year survival rate (5). The number of patients with a Fontan circulation is steadily increasing and was estimated to be 66 per million in 2020, of whom 55% are adults, 17% adolescents, and 28% children (3, 6).

Since the first reports of pioneering and lifesaving operations performed by Francis Fontan and Guillermo Kreutzer in the early 1970s (7, 8) in patients with tricuspid atresia, this procedure has been performed on many different types of congenital heart anomalies where a two-chamber circulation cannot be achieved.

The main features of the Fontan circulation are increased central venous pressure (CVP), low cardiac output, and lymphatic overflow with obstruction. There is an increasing awareness that chronic low flow in the Fontan circulation has a deleterious effect on cardiac function itself, but it also causes complications in nearly all organ systems, such as the lungs, brain, liver, bowels, kidneys, musculoskeletal system, and lymphatic system. The development of liver fibrosis and cirrhosis, known as Fontan-associated liver disease (FALD), is of special and growing concern regarding this young population.

Although liver fibrosis in a patient following Fontan palliation was first described in 1981, it has not been widely acknowledged until the last few decades (9, 10). The duration since Fontan completion has been associated with an increased stage of fibrosis (11–13). However, there are indications that *in utero* pre-Fontan insults may have implications for fibrosis development (14) and that FALD may start early, in childhood, with the development of subclinical fibrosis and cirrhosis into adolescence, which progresses to end-stage liver disease. As in other types of chronic liver disease, there is a risk of transformation to malignancy, particularly hepatocellular carcinoma (HCC), with an estimated prevalence of 0.18–1.3% in FALD (15, 16). Most reported cases of HCC have been reported in young adults, but single cases have been reported in early adolescence (15, 17).

The pathogenic mechanisms underlying FALD are still not fully understood. The course of development is known to be heterogeneous and variable among patients, with many unanswered questions about disease diagnosis, development, and prognosis. Most of the knowledge gathered to date is based on retrospective data from small populations. Prospective, longitudinal, and multicenter studies are urgently needed. This requires a consensus on how and when FALD should be diagnosed, and how disease development needs monitoring with regard to treatment optimization and surveillance for malignant transformation. Questions surround the process, timeline, and screening tools involved in forming this consensus.

Since early-stage FALD is characterized by subclinical development, imaging screening has become an important part of diagnosis and follow-up, together with clinical evaluations and scoring systems, histopathology, and serological data. This requires appropriate healthcare coordinated from pediatric to adult care, including not only cardiologists and hepatologists but also multiple subspecialists familiar with the consequences and pathophysiology of Fontan physiology.

## 2. Pathophysiology and outcome of the Fontan circulation

Single-ventricle circulation is a rare disorder and one of the most complex types of congenital heart disease (CHD). It constitutes less than 10% of CHD and is rather an umbrella category for diverse defects that all share one anatomic feature. Hypoplastic left heart syndrome is the most frequent type of defect, representing 1.4%–3.8% of CHD (18, 19). Other defects include unbalanced atrioventricular septal defects and hypoplastic right heart complexes with pulmonary atresia and tricuspid atresia.

The Fontan circulation is a definitive palliation for single-ventricle physiology and is currently achieved with stepwise sequential operations during the first years of life, with the final Fontan operation completed at 2–4 years of age.

**Figure 1:**
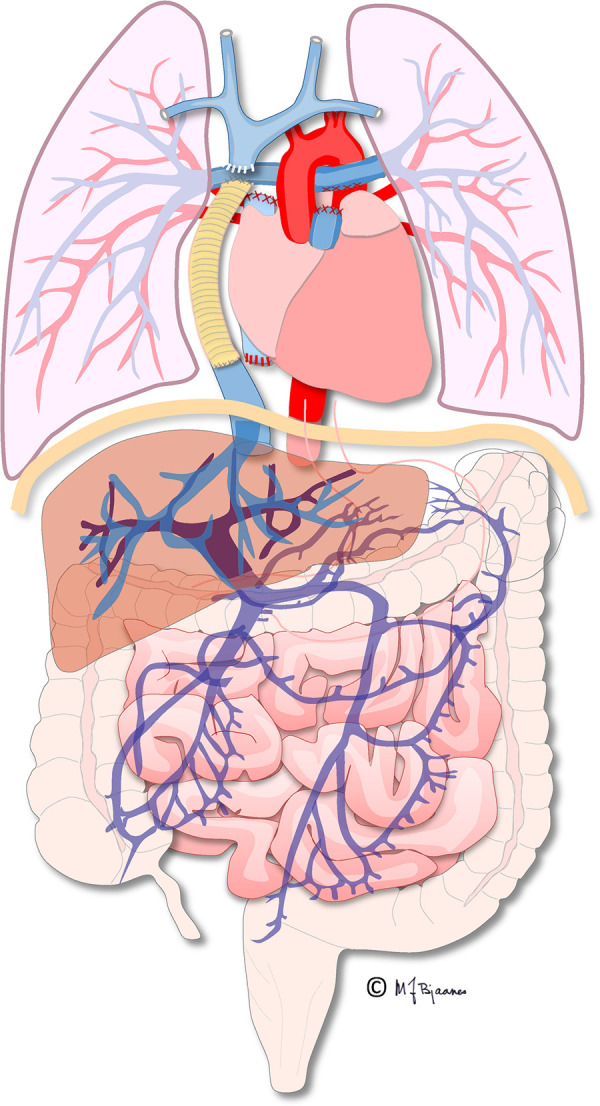
Illustration of the total cavopulmonary circulation with the extracardiac conduit connecting the inferior caval vein and the pulmonary arteries. Illustration created by Michael Bjaanes in collaboration with the Department of Pediatric Cardiology and the Department of Adult Congenital Heart Disease at Oslo University Hospital.

**Figure 2:**
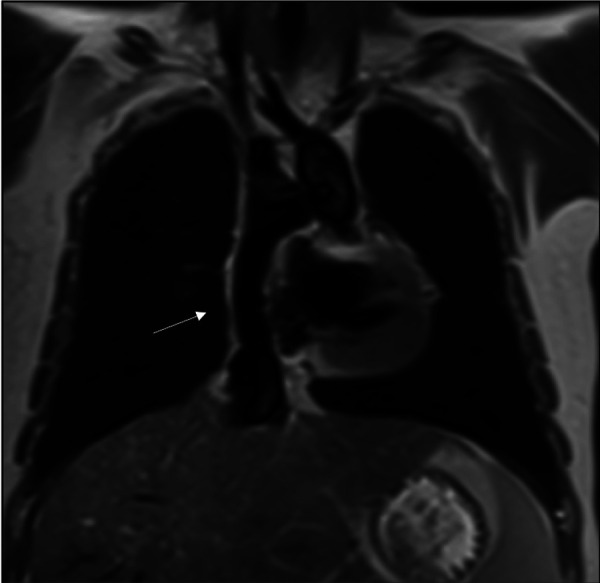
Cardiac MRI, T2 black-blood sequence in a coronal plane showing the extracardiac conduit (arrow) connecting the inferior caval vein to the pulmonary arteries.

The first procedure, performed shortly after birth, when necessary, ascertains blood flow and oxygenation to both the lungs and body. In addition, an unobstructed return of pulmonary and systemic venous blood is assured by a large atrial septal defect. In the second operation, the “bidirectional cavopulmonary connection” or “bidirectional Glenn procedure” is performed at approximately 3–6 months of age. Systemic venous return from the upper body is directly connected to the pulmonary arteries *via* the superior vena cava. In the final Fontan operation, pulmonary and systemic circulations are connected in series, now totally bypassing the subpulmonary ventricle directly channeled into the pulmonary arteries from the systemic veins by an intra-atrial “lateral” tunnel or currently by a synthetic extracardiac conduit, the “total cavopulmonary connection” (TCPC) (Figures 1, 2). This direct connection to the pulmonary arteries results in a slightly increased CVP to ensure blood flow into the lungs. Increased CVP limits the ventricular volume load reserve, which further results in a decreased cardiac output reserve (20).

Today, the operative mortality for the Fontan operation in most centers is relatively low (<3%), and the survival rate has increased considerably due to refinements in operative techniques, as well as pre-, peri-, and postoperative care (5). However, different organ complications develop over time, and most patients develop arrhythmias in the second or third decades of life (5, 21). On the other hand, there is wide functional variety in patients living with Fontan physiology, and there are patients with close to healthy cardiorespiratory fitness and without cardiac medication besides mandatory thromboemboli prophylaxis.

“Fontan failure” is the term used for a dysfunction originating from the heart itself or noncardiac complications including persistent congestion with edema, low cardiac output with limited exercise tolerance, an arrhythmia, protein-losing enteropathy (PLE), or plastic bronchitis (22, 23). This is associated with reduced quality of life and increased mortality, as well as limited options of treatment other than a cardiac transplant, which results in a considerably higher perioperative risk of mortality for such patients than other transplant candidates, especially in adults (24, 25). For a failing Fontan circulation, a total workup for an eventual transplantation should include an evaluation of liver disease comprising a physical examination, serological evaluation, and hepatic imaging.

## 3. Pathophysiology of FALD

**Figure 3:**
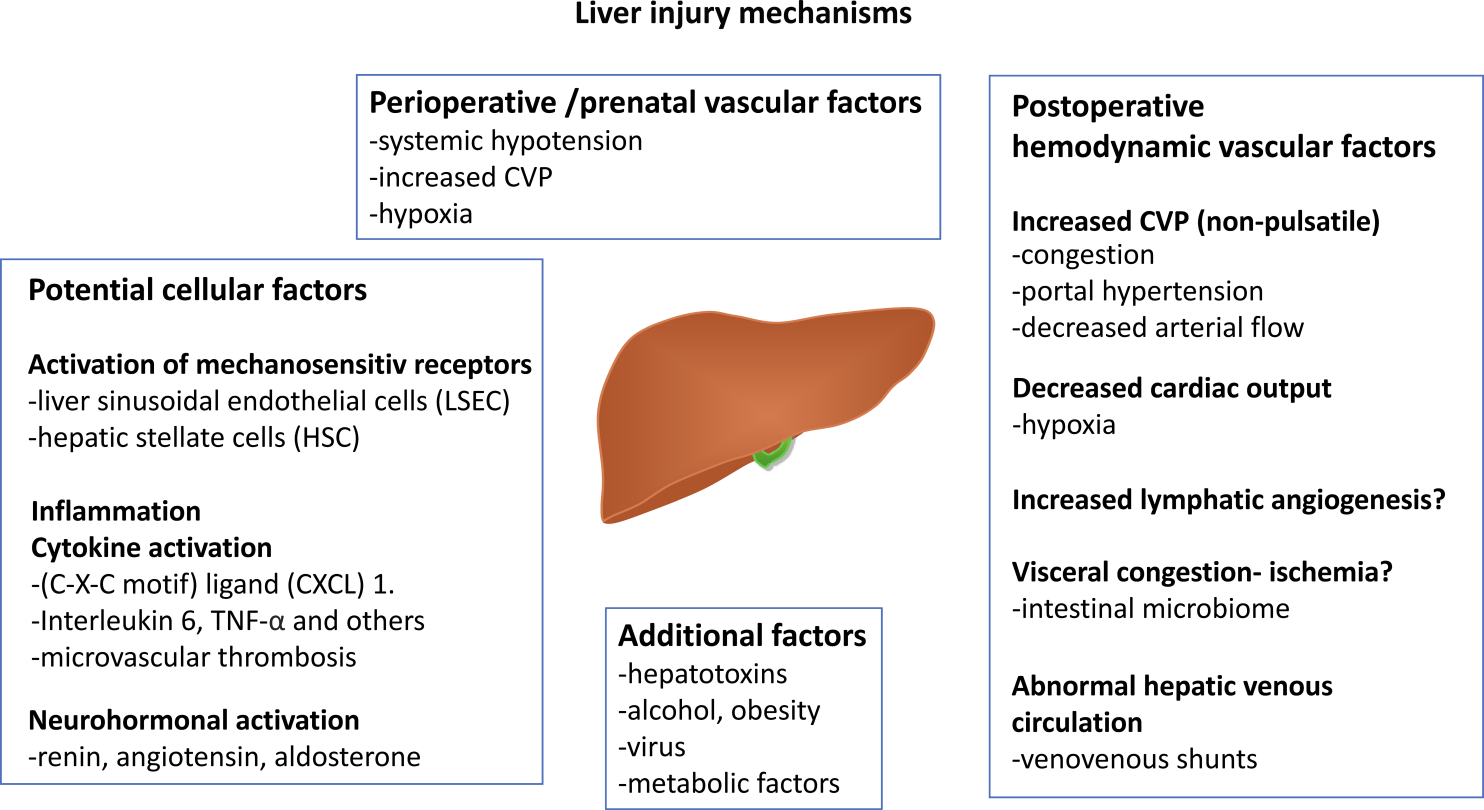
Figure displaying the most common proposed mechanisms influencing FALD development. Neutrophil chemotactic chemokine (C-X-C motif) and tumor necrosis factor-α (TNFα). A part of the illustration is created by Michael Bjaanes in collaboration with the Department of Pediatric Cardiology and the Department of Adult Congenital Heart Disease at Oslo University Hospital. FALD, Fontan-associated liver disease.

The mechanisms underlying the pathophysiology of FALD are multifactorial and not fully understood. Most of our knowledge is based on animal models or clinical/histological data from patient cohorts with other chronic liver diseases. The main factors that influence the FALD process are summarized in Figure 3.

### 3.1. Circulation

The chronic increase in CVP from the Fontan circulation has a negative impact on the liver (12). As portal venous inflow is regulated by mesenteric venous flow and the pressure gradient between the portal and hepatic veins, increased hepatic venous pressure hinders portal inflow to the liver. Furthermore, decreased cardiac output decreases portal venous inflow. The Fontan circulation with high CVP mimics a hepatic venous outflow obstruction, which causes arterialization of hepatic blood flow due to the “hepatic arterial buffer response.” This complicated regulatory mechanism ensures the autoregulation of hepatic inflow by the portal vein and hepatic artery (normally contributing 75% and 25%, respectively, of hepatic inflow) (26).

The congestion, with increased sinusoidal pressure, is subsequently transmitted to increase portal venous pressure. The hepatic artery buffer response will only operate at portal venous pressures <20 mmHg (27) and the development of portal hypertension (PHTN) above this threshold will reduce arterial inflow, resulting in arterial ischemia and hepatic necrosis. In this way, the liver in FALD is affected by two factors: reduced arterial and portal inflow and obstructed venous outflow.

In addition, aberrant Fontan circulation seems to facilitate the formation of intrapulmonary venovenous or arteriovenous collaterals that chronically further aggravate hypoxia (28, 29). Many anatomical single-ventricle physiologies result in different surgical technique-dependent flow characteristics of the Fontan connection. This might partly explain the individual differences in the severity of venous drainage from the liver and variability of liver damage (30).

### 3.2. Cellular

At the cellular level, hepatic congestion is suggested to cause a number of complex deleterious changes that interact with the development of liver fibrosis. Systemic venous hypertension transmitted to the hepatic veins leads to sinusoidal dilatation, hyperfiltration, and perisinusoidal edema with sinusoidal stretch, as shown in murine models of congestive hepatopathy and PHTN (31, 32). This perisinusoidal stress will trigger fibrotic transformation by activation of the mechanosensitive cell signaling pathway in liver sinusoidal endothelial cells (LSEC) and hepatic stellate cells (HSC) (26). In addition, stretching of HSC, also reported in murine models of congestive hepatopathy, induces fibronectin secretion and causes extracellular matrix assembly, which is an important step in fibrinogenesis (33, 34). Mechanical stretching disturbs LSEC function by decreasing the intrahepatic nitric oxide concentration. These changes hamper the diffusion of oxygen and nutrients and promote hepatocellular necrosis, atrophy, and fibrinogenic responses, which are more marked in the centrilobular zone (35). Furthermore, LSEC may play a role in early hepatic circulatory disturbance and the pathophysiology of PHTN in FALD with secretion of cytokines by LSEC and neutrophil chemotactic chemokine (C-X-C motif) ligand (CXCL) 1. CXCL1 further induces platelet aggregation with the formation of microvascular thrombosis, which increases PHTN and contributes to fibrosis (33, 34). Thromboembolic events are more frequently reported in patients with advanced FALD (36), and the efficacy of antithrombotic strategies, either with acetylsalicylic acid or warfarin, must be assumed to be influenced by the hepatic state.

Beyond the liver, other organs (i.e., kidneys and myocardium) can develop fibrosis in FALD (35). Interestingly, liver fibrosis was correlated with myocardial fibrosis in a study of young adult patients with a Fontan circulation and was associated with an increase in fibrogenic serum biomarkers, including matrix metalloproteinases and tissue inhibitors of metalloproteinases, suggesting a profibrotic environment and deleterious effects on end-organ function (36).

### 3.3. Inflammation and biochemical markers

The role of inflammatory mediators has been previously reported from small studies on patients with heart failure (37). Recently, a prospective study of 44 stable patients operated with a Fontan circulation revealed increased inflammatory biomarkers and complement and systemic cytokines, including interleukin 6, growth derived factor-15, tumor necrosis factor-α, and *β*2-macroglobulin (38).

The relationship between lymphatic overflow, congestion, and end-organ damage, such as liver fibrosis and PLE, is still not well understood. Increased lymph angiogenesis in the liver has been shown to contribute to dilatation of the hepatic sinusoids, and poor lymph drainage may likely contribute to aggravating liver dysfunction, tissue inflammation, and fibrosis (39). Rodriguez de Santiago et al. found a relationship between advanced liver disease assessed using imaging, PLE, and markers of inflammation and intestinal permeability in a small prospective adult Fontan study (40).

Neurohormonal activation seems to play a role in FALD, where serum renin, aldosterone, and angiotensin are shown to be increased in patients with Fontan physiology, and where angiotensin 2 promotes the secretion of collagen 1 (35, 38).

Finally, heart failure and visceral congestion alter intestinal perfusion with ischemia and increase intestinal permeability, with bacterial translocation events and systemic inflammation (41, 42). Recent studies have shown that the intestinal microbiome may play an important role in congestive hepatopathy, but its role in the Fontan circulation has not been investigated (41).

## 4. Clinical presentation

FALD most often exhibits subclinical development during the early phases. This demonstrates the need for disease detection screening. Clinical and laboratory findings vary according to the disease stage, and in many pediatric patients, they may be subtle, resulting only in hepatomegaly due to congestion (11, 43, 44). Even in the advanced stage of FALD with established but compensated cirrhosis, the clinical symptoms may be absent or present with more nonspecific symptoms, such as anorexia, fatigue, and weight loss (43, 45, 46). Importantly, the presence of ascites in the Fontan circulation can make it difficult to diagnose whether there is a combined cardiac and hepatic dysfunction or the presence of PLE. Pediatric patients with a Fontan circulation and ascites may have other abnormalities such as obstruction of the Fontan conduit. To differentiate ascites of hepatic from cardiac origin, brain natriuretic peptide (BNP) can be used to exclude a cardiac origin (47).

In late-stage and decompensated liver disease, ascites, jaundice, variceal bleeding, and hepatic encephalopathy may play a role and suggest the presence of PHTN, which is associated with adverse outcomes in the population with a Fontan circulation (48, 49). Identifying PHTN is important for optimizing clinical management, that is, screening for variceal bleeding and diuretic medication, and may implicate health outcomes and the decision for liver transplantation (49).

Variceal bleeding can be reported after the Fontan procedure, with a variable incidence ranging from 9.3%–38% (50, 51). In children, the prevalence of varices is even lower (about 9%), which suggests that its development is a late complication (51). Because of the high risk of mortality of this complication (15%–20%) (52), upper gastrointestinal endoscopy is commonly recommended for adult patients with cirrhosis or signs of PHTN. Imaging is not recommended as part of the screening for varices, although they can be observed with cross-sectional imaging (48).

Laboratory findings are characteristically only slightly affected by liver congestion. There are often increased aminotransferases in approximately 30% of cases accompanied by a slight increase in alkaline phosphatase (ALP), gamma-glutamyl transferase (GGT), and indirect bilirubin, in addition to a prolongation of prothrombin time (53–55). Decreased serum albumin is noted in 30%–50% of patients with chronic heart failure (56).

## 5. Hepatocellular carcinoma

HCC is a rare complication with an estimated prevalence of 0.18%–1.3% in the population with a Fontan circulation (15, 16). The youngest patient reported was 12 years of age at the time of diagnosis, while the median age after Fontan completion was 22 years in two large retrospective multicenter studies (15, 57). Only 50% of patients were symptomatic at the time of diagnosis, presenting with symptoms including jaundice, abdominal pain, ascites, dyspnea, hematemesis, and fever. In a multicenter retrospective study including 54 patients with HCC who underwent a Fontan operation, the mean age was 30 years and the median tumor size at diagnosis was 4 cm (range, 1–22 cm) (57). Survival was poorer if patients had clinical symptoms at diagnosis and if the tumor size was >4 cm; the cumulative survival rate was only 50% after 1 year (57). An important feature found was that 50% of patients did not have cirrhosis when diagnosed with HCC, indicating that having established cirrhosis is not a requirement for HCC development in patients with a Fontan circulation. This has also been confirmed in smaller studies on such patients. Mazzarelli et al. revealed that HCC was registered in three patients without an association with the degree of hepatic cirrhosis, which was also confirmed in a meta-analysis by Rodriguez de Santiago et al. (58, 59). Longitudinal studies are needed to identify the risk factors for developing HCC and to find a suitable surveillance strategy to identify HCC at an early stage.

## 6. Diagnosis

As mentioned previously, the diagnosis of FALD is a clinical challenge. However, the true incidence of FALD is not known and likely under-recognized since there is no consensus on a uniformly accepted definition of the disease. There are different definitions of FALD based on serological biomarkers, combined radiological and serological scores, and histological and clinical evaluations. A recent expert consensus statement from an association of several leading societies focused on CHD and the care of patients with a Fontan circulation proposed the following definition of FALD: “The broad spectrum of liver disease and its consequences, attributable to Fontan hemodynamics. FALD includes varying is degrees of hepatic fibrosis, compensated and decompensated cirrhosis, focal nodular hyperplasia [FNH], laboratory evidence of hepatic injury or impaired synthetic function, and hepatocellular neoplastic lesions” (60). This indicates that FALD can be present even without Fontan failure.

Liver biopsy remains the gold standard for the diagnosis of adult noncongestive liver disease. Nevertheless, this is more complicated in the case of FALD because there may be sample errors due to the heterogeneous nature and patchy distribution of fibrosis (20). With the invasive procedure, there is a potentially increased risk of bleeding due to anticoagulant medication and high CVP, and biopsy is therefore not suitable for routine longitudinal follow-up. In the pediatric population, sedation and general anesthesia are often required.

Consequently, noninvasive diagnosis and staging could be an attractive alternative, but with different limitations. Imaging alone cannot reliably detect early stages of fibrosis. Ubiquitous liver congestion in FALD can present with confounding imaging features that resemble advanced cirrhosis. Congestion is also a confounder for noninvasive imaging of fibrosis biomarkers, such as elastography (61, 62). In addition, serological markers of liver function do not correlate with liver fibrosis severity (13, 63).

Ultimately, the definition of advanced FALD with cirrhosis consists of a combination of supportive findings from clinical, laboratory, and imaging parameters of the liver and abdomen (presence of varices, portosystemic collaterals, ascites, and splenomegaly) (13, 56).

### 6.1. Serological markers and clinical scoring systems

Most serological tests used to estimate liver fibrosis in other chronic liver diseases have been evaluated in FALD, although most studies did not include data on histology as the gold standard nor considered the intrinsic limitations of these noninvasive parameters (64–66).

In compensated FALD, hepatic serological markers are normal or slightly affected and increase over time, including a mild increase in the GGT level, which is the most common finding (50, 67) often combined with a mild increase in unconjugated hyperbilirubinemia.

Sometimes, a combination of mild elevations of aspartate aminotransferase and alanine aminotransferase is observed in mild congestion and may be accompanied by a moderate elevation of ALP (68). Elevated prothrombin time and a prolonged International Normalized Ratio (INR) may also be found but are rarely related to the degree of fibrosis (69).

It is not until the more advanced stages of fibrosis and cirrhosis that serological markers are markedly affected with an additional decrease in platelet count as a sign of PHTN and splenic consumption (70). In the case of HCC development in the Fontan circulation, an increase in *α*-fetoprotein may be seen in 74%–80% of patients (57, 59). Low serum albumin levels may occur secondary to PLE, nephropathy, and malnutrition. Elevated liver test results may stem from cardiac dysfunction (especially regarding GGT), medical treatment (antiarrhythmic drug toxicity), surgical procedures, or cardiac collapse (71).

#### 6.1.1. Laboratory scoring systems

Laboratory scoring systems have not been validated for the population with FALD but might aid in the evaluation of cirrhosis as a supplement to imaging findings and clinical assessment. Many different systems have been used with different profiles and limitations in the setting of FALD, with modest discriminatory power in identifying patients with severe fibrosis (Table 1).

**Table 1:** Laboratory scoring systems.

Scoring system	Variables	Prediction of fibrosis in FALD
AST/ALT ratio	AST, ALT	Difficult to evaluate early fibrosis since a mild increase in these serum markers is common due to congestion
APRI	AST, platelet count	Dedicated to scoring hepatitis C not validated in FALD. Decrease in platelet count is associated with advanced disease
FIB-4	AST, ALT, platelet count, age	Performs less well for FALD patients since the majority are <40 years
MELD	Bilirubin, creatinine, INR, sodium	Not appropriate in FALD patients with anticoagulant therapy
MELD-IX	Bilirubin, creatinine	Correlates to biopsy-proven FALD fibrosis. Potential role in predicting outcome for transplantation heart vs. heart–liver
Forns index	GGT, cholesterol, platelet count, age	Takes into account GGT which is also commonly increased in congestive hepatopathy
Pohl score	AST, ALT, platelet count	Dedicated to scoring for hepatitis C not validated in FALD. Decrease in platelet count is associated with advanced disease
Cirrhosis discriminant score	GGT, AST, ALT, INR, upper limit of AST, platelet count, age	Dedicated to scoring for hepatitis C not validated in FALD

ALT, alanine transaminase; AST, aspartate aminotransferase; APRI, aspartate aminotransferase to platelet ratio index; FIB-4, fibrosis-4; MELD, model for end-stage liver disease; MELD XI, model for end-stage liver disease excluding the international normalized ratio; FALD, Fontan-associated liver disease; INR, International Normalized Ratio; GGT, gamma-glutamyl transferase.

Of these tests, the aspartate aminotransferase to platelet ratio index and fibrosis 4 score are dedicated for patients with hepatitis C and fibrosis development, and indicate PHTN, but have not been proven useful in FALD (72). The Forns index (including age, GGT, cholesterol, and platelet count) has shown a correlation between the duration of the Fontan circulation and signs of cirrhosis shown on computed tomography (CT) (64).

Other scores involving INR, albumin, and bilirubin, like the Child–Pugh score or “the model for end-stage disease” (MELD), may not reflect liver function properly in the population with FALD since these patients commonly receive anticoagulation therapy. In this regard, “the model for end-stage disease, excluding INR” (MELD XI) seems more promising (51, 73). MELD XI has been shown to correlate with biopsy-proven liver fibrosis, but a specific cut-off for severe liver fibrosis has not been identified (74). MELD XI may have a role in predicting the outcome of transplantation and need for a combined heart–liver transplant vs. an isolated heart transplant (75).

### 6.2. Liver biopsy—histopathology

Traditionally, liver biopsy has been performed to stage different types of chronic liver disease and is considered the diagnostic gold standard. The contribution of liver biopsy, performed *via* a transjugular or percutaneous approach in the population with a Fontan circulation, to assess liver fibrosis and cirrhosis is not clear, and findings are contradictory. Several limitations of performing a biopsy are present in the setting of FALD; sample errors are frequent owing to the particularly patchy distribution of fibrosis. Because it is an invasive procedure, there is an increased risk of bleeding. In Fontan physiology, this is of special concern when performing a biopsy given cardiac and liver dysfunction with increased CVP and the need for systemic anticoagulation therapy (76). However, in a recent pediatric study, transcutaneous biopsy-related complications were similar to those in other pediatric cohorts (77). Performing transjugular biopsies during cardiac catheterization has an advantage over percutaneous biopsies in that they are safer with regard to bleeding and allow for the simultaneous assessment of hemodynamic data. Nevertheless, they are equally challenging with the risk of producing unreliable samples that overestimate the grade of fibrosis, since sampling is mainly performed more centrally close to the liver veins, where the disease is thought to be more pronounced (69).

#### 6.2.1. Histological scoring

FALD exhibits a distinctive histological pattern of congestive hepatopathy. In FALD, periportal, centrilobular, and sinusoidal fibrosis coexist, unlike other inflammatory diseases such as viral hepatitis or alcohol-induced liver disease, where there is centrizonal portal-based fibrosis secondary to hepatic congestion (78). The etiology of portal fibrosis in FALD is not fully understood, and histopathological reports suggest a contribution of injury from pre-Fontan insults (14).

This disease pattern makes traditional histopathological scoring systems for chronic liver disease, such as the METAVIR or Ishak score, focusing on portal fibrosis, inadequate for assessing the whole extent of disease in FALD. In a study of adult patients with congestive hepatopathy (only two with a Fontan circulation), a new scoring system for congestive hepatic fibrosis was developed. Taking into account central and portal fibrosis, the “congestive hepatic fibrosis score” was suggested (79). Furthermore, studies have combined two of these scoring systems in the “modified Ishak congestive hepatic score,” which accounts for both portal and early central fibrosis with a more refined scale that could be more representative of FALD histology (80). Although these scores have been used for FALD in several later studies, prospective data are lacking regarding their prognostic value for monitoring FALD and predicting clinical outcomes (46, 65, 69, 80, 81).

*Liver biopsy in suspected FALD should be performed after careful consideration and is mainly indicated in three possible clinical situations:* (*1*) *as part of the follow-up, (2) before heart transplantation, or (3) to characterize liver nodules*.

(1) Biopsy as part of FALD follow-up*:* Some centers perform liver biopsy as part of their FALD surveillance program; however, most available data come from retrospective adult studies. A review of 67 young, predominantly asymptomatic adolescents and young adults found a moderate correlation between the grade of fibrosis and time from TCPC surgery, but no correlation with hemodynamic risk factors (20). In another retrospective study of 74 biopsies from asymptomatic, pediatric, and adult patients with Fontan physiology, a median age of 17.7 years (range, 7.2–26.9 years) and various degrees of portal fibrosis in 93% of patients were revealed (69). Other studies have revealed a general presence of centrilobular or sinusoidal fibrosis with a portal fibrosis prevalence of 82%–93% and universal centrilobular fibrosis with an advanced fibrosis prevalence of 39%–78% (54, 74, 76, 78). Correlations with clinical outcome in 68 patients were studied by Wu et al., and no correlation was found between histology and patient death or transplant-free survival (76).

However, it is important to keep in mind that a small focal core biopsy is not representative of FALD given its heterogeneous distribution. This is illustrated in a study of FALD liver explants from 15 patients, where explant histology revealed higher grade fibrosis than the transjugular biopsy performed shortly before death in 40% of patients (82).

In conclusion, the utility of biopsy for staging fibrosis in FALD is still under debate, given its risks and poor correlation with clinical outcomes.

(2) Biopsy in the decision workup for a transplantation: Liver biopsy is part of the investigation to determine whether heart transplantation should be associated with liver transplantation (31, 83). In this scenario, consensus is not universal, especially regarding the capability of histology to predict whether the patient will survive with a heart-only transplant or to influence the best timing for surgery (77).
(3) Biopsy to characterize liver nodules: Focal liver lesions of different sizes occur frequently in patients with a Fontan circulation, and imaging findings can be more difficult to interpret than in other types of chronic liver disease. Biopsies remain important for the evaluation of liver nodules in FALD. This is discussed further in a later section.

### 6.3. Imaging

FALD imaging features are distinct from those of other etiologies of chronic liver disease. Over its course, FALD may be visualized, and to some extent, fibrosis can be quantified with different radiological techniques such as abdominal ultrasound (US), CT, and magnetic resonance imaging (MRI).

Common findings with all techniques involve an enlarged liver with bulging contours and a dilated inferior vena cava and hepatic veins due to elevated CVP. Venovenous shunts may also be present. The heterogeneous distribution of fibrosis is also represented by multiple peripheral nodules of different sizes in both early and late disease stages. With time, cirrhosis develops and atrophy of the right lobe may be observed with a nodular surface. Typically, a patchy and uneven distribution of fibrosis or cirrhosis is seen (63).

#### 6.3.1. Ultrasonography with Doppler examinations

Although operator-dependent, US is an easy bedside technique suitable for the first evaluation of the liver parenchyma. US allows for the assessment of hepatic blood flow and can also provide an overview of the abdominal organs. It screens for signs of liver-associated complications, such as splenomegaly and ascites. However, US cannot reliably detect early fibrotic changes in the liver, but it can be important in the serial follow-up of more gross parenchymal changes combined with hemodynamic changes. In the early phase of congestive hepatopathy, the liver appears enlarged with slightly heterogeneous echogenicity, which becomes more pronounced over time. Additionally, US may identify hyperechoic nodules of variable sizes. Some nodules can also be hypoechoic, more difficult to visualize in heterogeneous liver tissue, and better visualized with cross-sectional imaging (84–86).

Spectral and color Doppler examinations are important for evaluating the flow patterns of hepatic vessels (63). Assessment of portal venous mean flow velocity has been proposed as an indicator of PHTN in Fontan physiology (87).

Contrast-enhanced ultrasound (CEUS) using the intravenous injection of microbubbles is used to characterize the benign or malignant contrast enhancement pattern of a nodule (88). The US contrast agent SonoVue ® (sulfur hexafluoride; Bracco, Milan, Italy) is used off-label in Europe for intravenous applications in children younger than 18 years of age. The experience of using CEUS for patients with a Fontan circulation is low because they often have collateral circulation with right-to-left shunts, which is considered a contraindication to CEUS in Europe (89) but not in the United States (90, 91).

#### 6.3.2. Computed tomography and magnetic resonance imaging

Cross-sectional techniques, such as CT and especially MRI, can provide more detailed and accurate complementary information on the liver parenchyma and all abdominal organs. Using intravenous contrast agents, liver vascularization in different phases and nodular enhancement patterns can be well visualized. However, these techniques are expensive and have different strengths and weaknesses. CT is faster and more accessible than MRI; however, it unfortunately involves ionizing radiation. Serial CT examinations should be avoided in children and young adults because they are more sensitive to radiation. This is particularly important in patients with congenital heart disease, who have a higher risk of developing cancer (92). Hepatic MRI is preferable; however, MRI is more expensive and requires a longer examination time. Furthermore, MRI may not be possible owing to incompatible cardiac devices; therefore, in these cases, CT of the liver is recommended.

**Figure 4:**
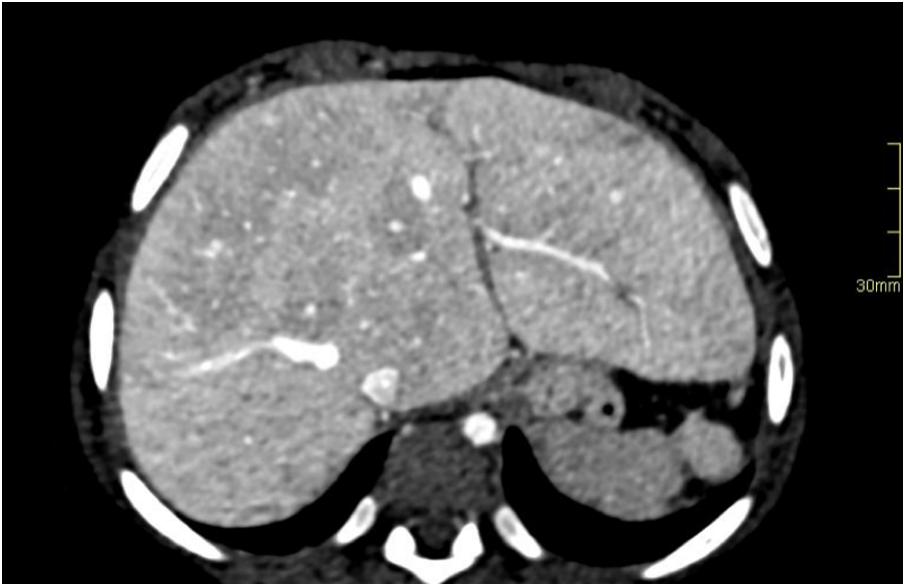
Hypoplastic left heart syndrome with Fontan circulation in a 4-year-old girl with failing Fontan. Abdominal CT with i.v. contrast, portal venous phase, in an axial plane reveals hepatomegaly with round hepatic borders and perisinusoidal edema. A biopsy performed pretransplantation, revealed fibrosis grade 3–4. Note the small spleen due to previous infarction.

**Figure 5:**
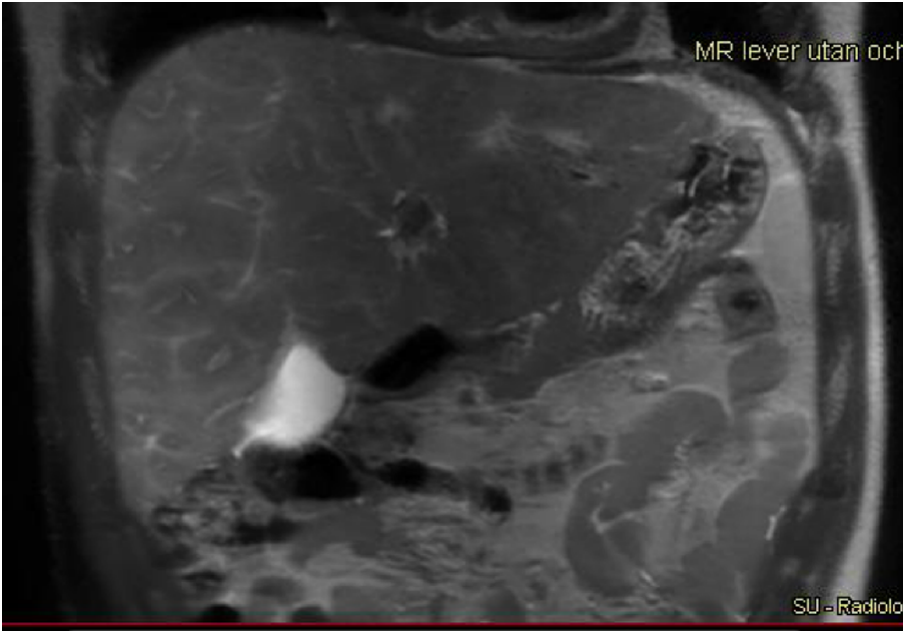
A 17-year-old boy with hypoplastic left heart complex operated with a Fontan circulation. Abdominal MRI, a coronal T2-weighted image, reveals the typical heterogenous distribution of the liver disease with irregular signal, perisinusoidal edema, in the right liver lobe with a more homogeneous signal in the left lobe.

**Figure 6:**
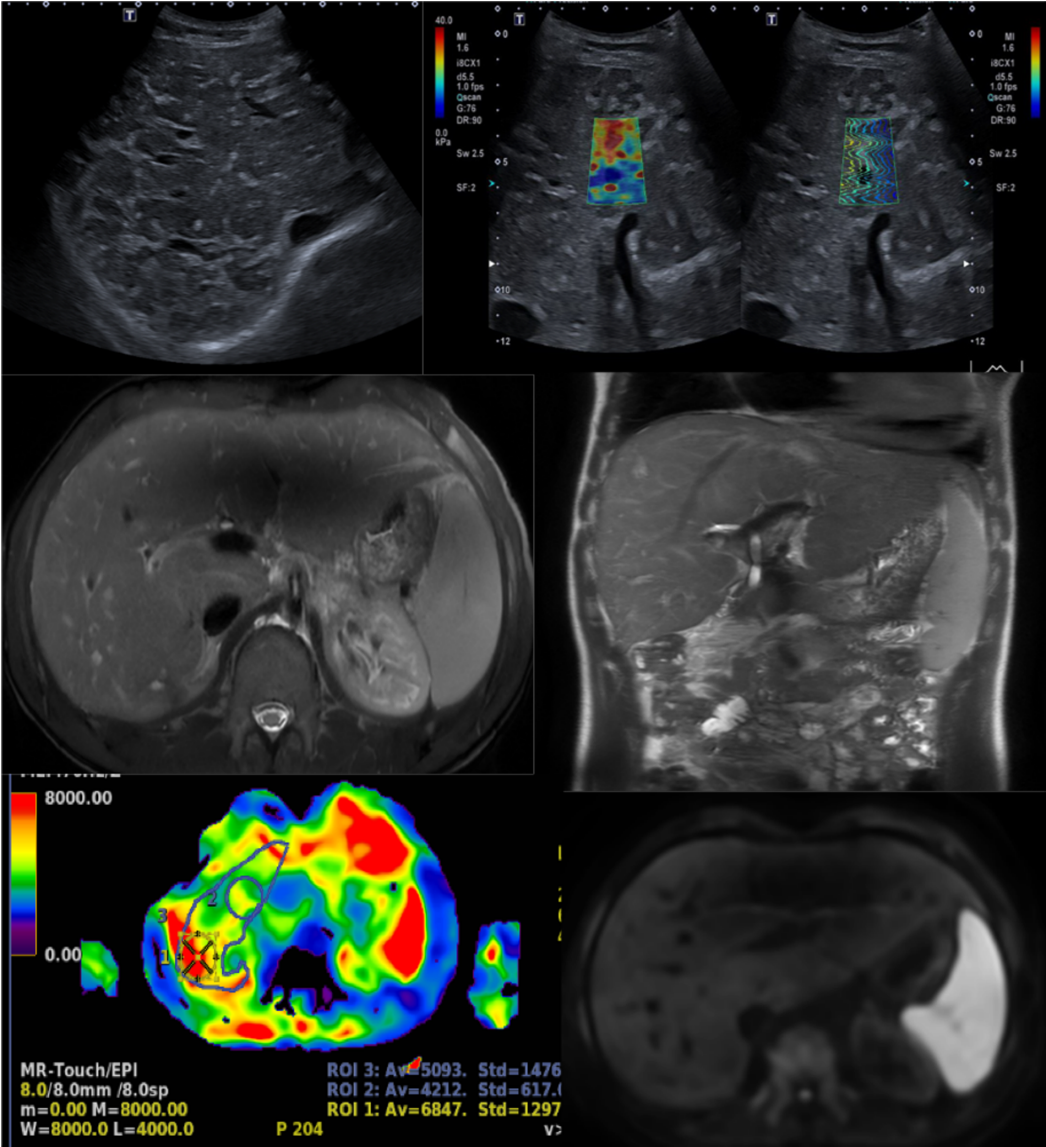
A 17-year-old boy with a hypoplastic left heart complex and Fontan circulation (same patient as in Figure 5). Clinical workup revealed a slightly increased GGT and a newly developed reduced exercise capacity. Ultrasound reveals severe heterogeneous echogenicity (upper left) and increased values of elasticity of 25 kPa (upper right). Abdominal MR T2-weighted coronal image (middle right), with increased size right liver lobe. The liver length is 20 cm and there is a splenomegaly measuring 17 cm in length. Corresponding axial T2-weighted image reveals the reticular peripheral pattern (middle left). Heterogenous diffusion (lower right). MR elastography in an axial plane, measuring increased values of 5.1 KPa (lower left). GGT, gamma-glutamyl transferase.

**Figure 7:**
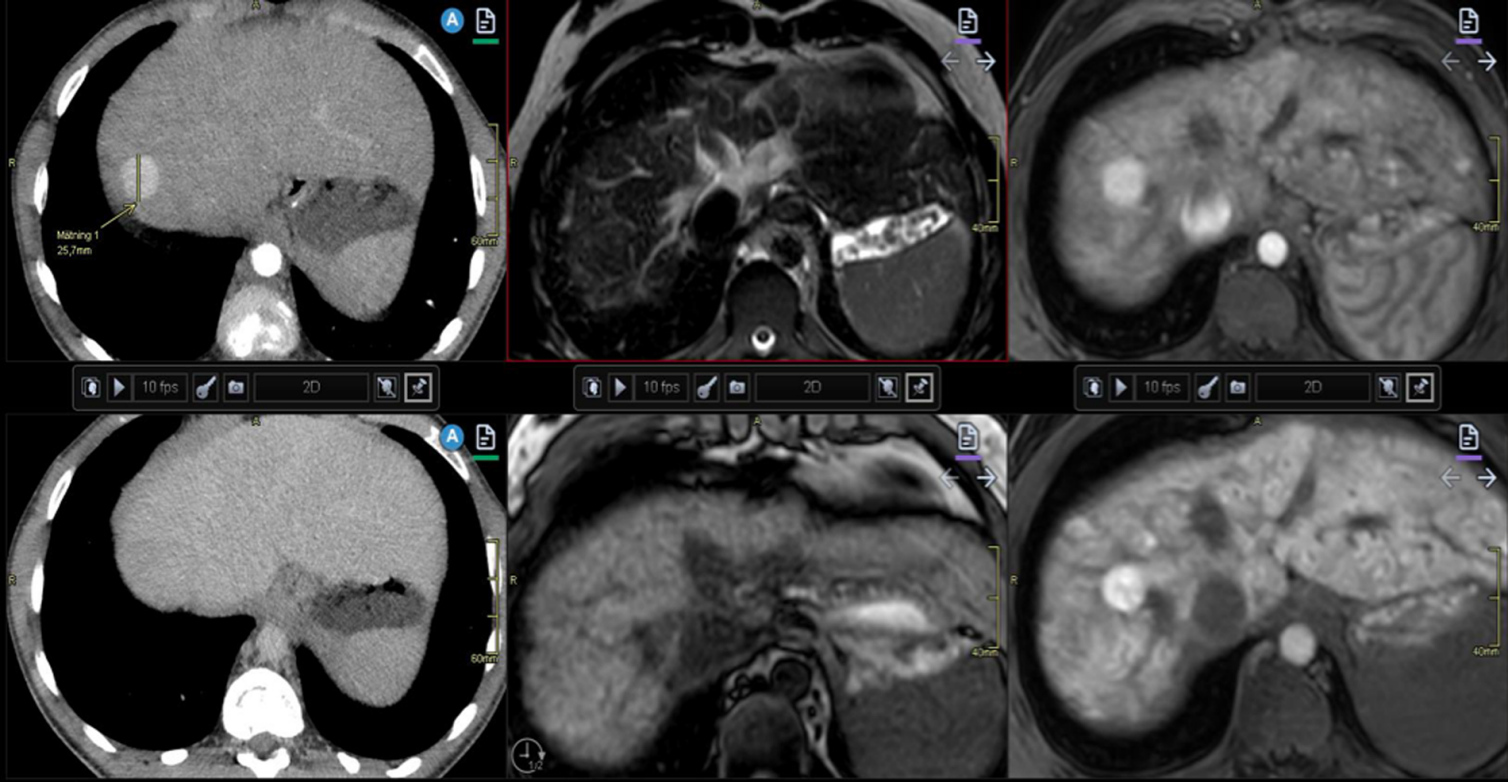
Illustration of liver nodules in a 20-year-old man with pulmonary atresia and a Fontan circulation. Increased hepatic serological marker, and alkaline phosphatase was observed. Multiphase contrast-enhanced CT revealed a nodule of 2.5 cm in diameter in segment eight with arterial enhancement (upper left) and apparent washout on portal venous phase (lower left). Follow-up MRI with hepatocyte-specific contrast displays low signal on T2-weighted (upper middle) and isointense signal on T1 (lower middle). Arterial enhancement (upper right) and slightly heterogeneous hyper-enhancement on late hepatocyte phase (lower right). Note the heterogenous congestion and multiple small surrounding nodules in this late phase. These are both benign and malign features. Biopsy in this case was considered difficult due to the high position of the nodule under the diaphragm. Close monitoring was recommended. CT, computed tomography; MRI, magnetic resonance imaging.

The preference for MRI over CT in the imaging of liver disease is increasing because of advances in tissue characterization, the use of hepatobiliary contrast agents, blood flow quantification, and MR elastography (MRE) (93). Severe fibrosis is observed with CT and MRI as surface nodularity, blunt liver margins, a smaller or atrophic right lobe, and hypertrophy of the caudate and left lobe (Figures 4–6). Extrahepatic manifestations such as ascites, gastroesophageal varices, and splenomegaly may also be present (94, 95). A standard MR protocol for the liver includes T1- and T2-weighted sequences, gradient echo sequences in and out of phase, and diffusion-weighted imaging. Additionally, the liver's vascular phases can be studied with dynamic gadolinium contrast-enhanced sequences (gadoteric acid) or by using a gadolinium-based hepatocyte-specific contrast agent (gadoxetic acid) (63). Gadoxetic acid is partly excreted by the liver and complements enhancement information on the late hepatobiliary phase. The T2-weighted images frequently exhibit a diffusely increased liver signal intensity that might reflect the degree of congestion and patchy patterns on diffusion-weighted imaging (Figure 6) (43, 63, 96). For the Fontan circulation, the vascular contrast enhancement pattern on CT and MRI of the liver demonstrates a distinct peripheral, reticular, and patchy enhancement pattern during the late arterial and portal venous phases, which equilibrates with the background signal of the liver during the delayed phase (97). This configuration is probably due to a delayed wash-in of the contrast material into the congested liver (Figures 4 and 5). Heterogeneous general hypo-enhancement in the hepatobiliary phase with MRI may reflect decreased hepatic function (Figure 7) (98).

In conclusion, conventional morphological imaging features of US, CT, and MRI have proven to be useful in characterizing liver fibrosis, but these modalities cannot provide sufficient information regarding the development of early-stage fibrosis as reliably as tissue analysis (46, 94). Imaging findings are commonly not observed until the degree of fibrosis is moderate to severe due to the mixed and competing effects of hepatic congestion (swelling) and fibrosis (shrinking) (63, 98, 99).

#### 6.3.3. Lymphography

**Figure 8:**
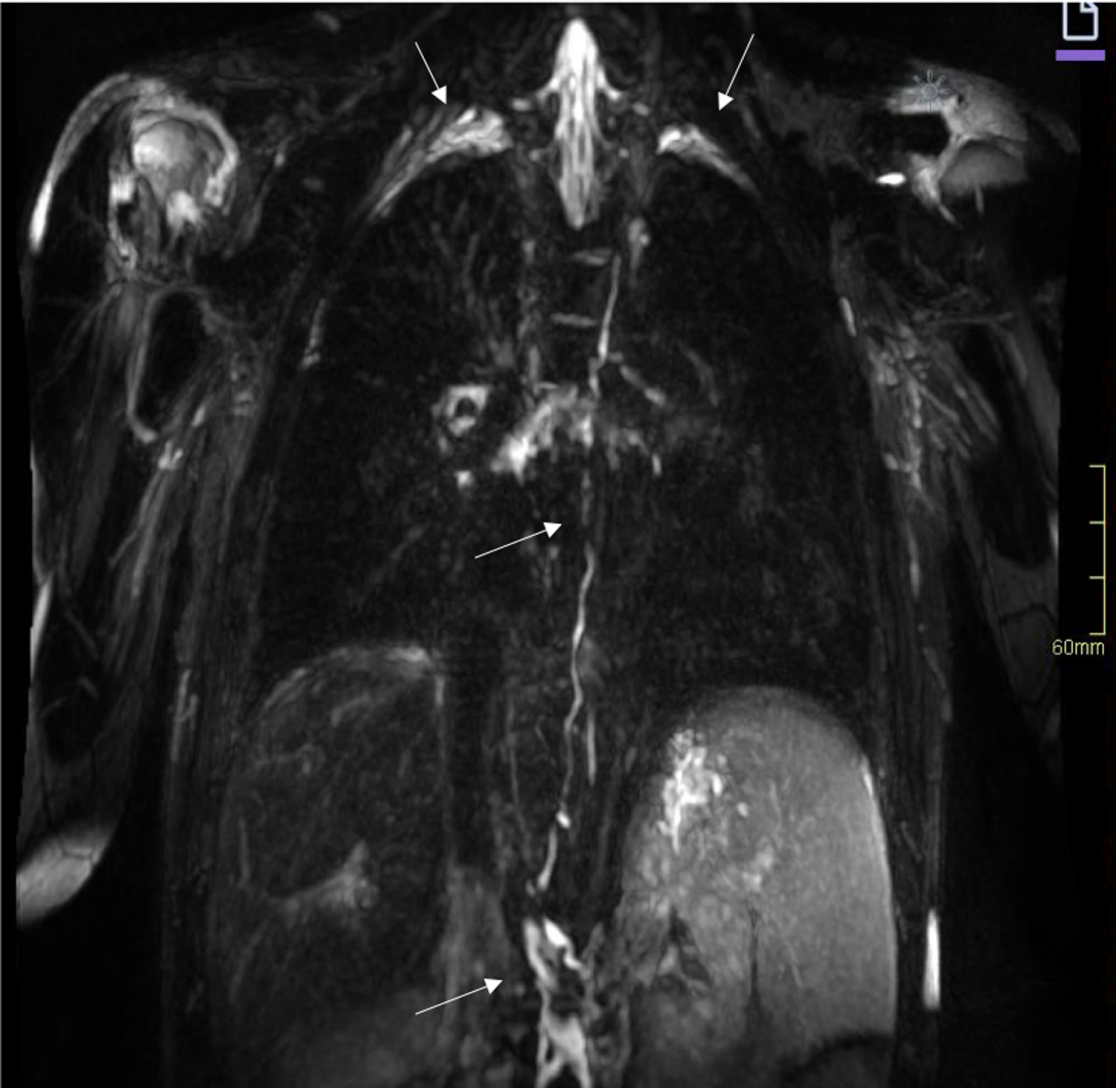
MR lymphography of the thorax in a coronal plane with maximum intensity projection, of the same patient as in Figure 6, reveals a tortuous cisterna chyli and thoracic duct (arrows) and lymphedema in the perihilar region as well as small amounts of pleural effusion over the apices of the lungs (arrows). MR, magnetic resonance.

**Figure 9:**
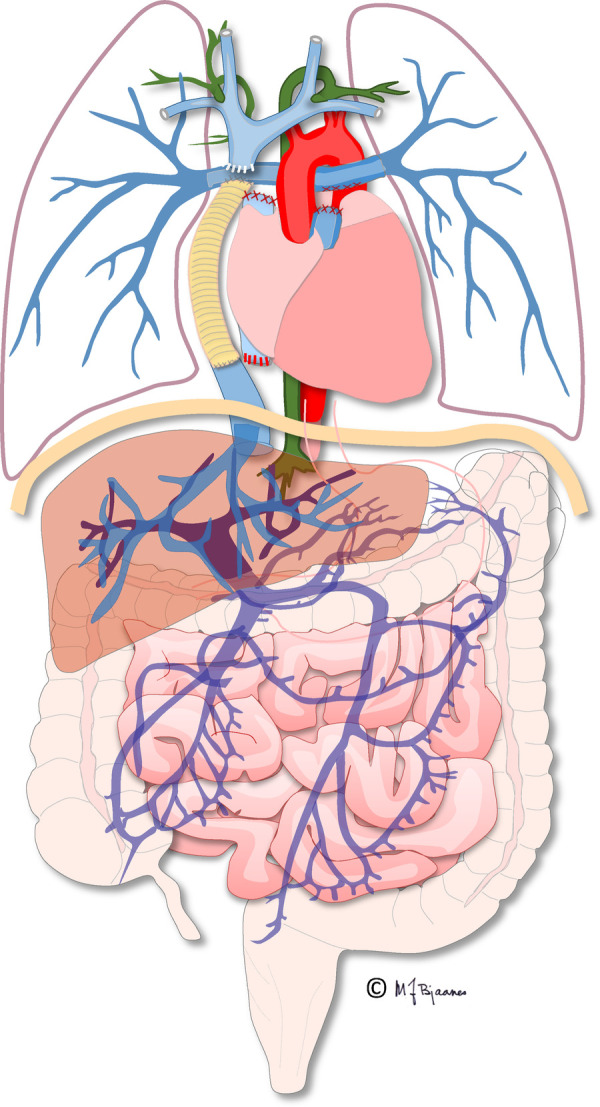
Illustration of the total cavopulmonary circulation including the thoracic duct (in green). Illustration created by Michael Bjaanes in collaboration with the Department of Pediatric Cardiology and the Department of Adult Congenital Heart Disease at Oslo University Hospital.

MR lymphography of the thorax and abdomen is an important part of the total imaging and hemodynamic cardiac workup and could be important in the diagnosis and staging of FALD, although its current role for this indication requires further evaluation. MRI of the lymphatic system is based on either 3D heavily T2-weighted sequences without contrast enhancement or dynamic contrast-enhanced MR lymphography, which can identify patterns of lymphatic vascular architecture (Figures 8, 9) (100–102). In addition, the use of conventional lymphatic angiography or intrahepatic MR lymphography in several studies has been reported (103, 104). With MRI, lymphatic congestion in the liver and mesentery might be displayed as a perivenous increased signal and dilation of the cisterna chyli continuing with the thoracic duct. Furthermore, dynamic contrast-enhanced imaging can reveal leakage sites responsible for PLE (101, 105).

#### 6.3.4. Quantitative imaging techniques

Supplementary imaging techniques enabling the quantification of fibrosis, edema, inflammation, and fat content in the liver are desirable and under development as current research topics.

*Elastography* displays the elastic restoring forces in the liver tissue by shear wave deformation and can be performed using US or MRI (105).

*US elastography* is commonly used to stage hepatic fibrosis and cirrhosis in noncongestive chronic liver disease (106–108). However, the accurate use of elastography in hepatic congestion is not clear, as congestion may confound liver stiffness measurements. There is increasing experience with the serial follow-up of elastography for patients with FALD, especially in adults. Typically, these patients have increased stiffness due to different proportions of congestion and fibrosis already reported in the postoperative period after TCPC (Figure 6). High elastography values are often obtained within the range found for advanced cirrhosis in adult chronic liver disease (109, 110); however, there is no clear correlation with fibrosis based on the histology of adult and pediatric patients (46, 62, 111–113).

MRE uses shear wave deformation technology as in US. There is still little evidence of the correlation between MRE and fibrosis staging in FALD; however, a small study with adult patients revealed a strong correlation between liver stiffness and histology and time since the Fontan surgery (61). Additionally, in a recent small study, MRE compared with transjugular liver biopsy in 49 patients reported a moderate correlation with histologic fibrosis grade (80). Finally, the presence of nodules in the Fontan liver has been associated with increased US or MRI stiffness and vice versa (84, 85, 114).

Along with new measures in US elastography, shear wave dispersion has been reported to be related to tissue viscosity and thus seems to reflect the components of inflammation, edema, and necrosis (115–118). In addition, the US attenuation imaging coefficient is an additional tool that enables the surrogate estimation of fat content in the liver (108, 119).

In MRI, MR fat fraction and relaxometry techniques have been reported to have potential value in the diagnosis of FALD related to Fontan pressures and PHTN (120–123) but are still under research.

There is a complex relationship between liver stiffness, grade of fibrosis, Fontan pressure, and Fontan failure, as illustrated by the above studies. Different techniques have several important limitations that may influence disease interpretation. US elastography will only display a small region of the right liver lobe, which might not be representative of a patchy disease. MR elastography using the 2D technique and 2D MR relaxometry will display a slice of the liver in an axial plane and will subsequently display more tissue properties, but not the whole liver. Nevertheless, it is possible that liver stiffness measurements performed under standardized conditions over time in individual patients may be valuable biomarkers of FALD development, and unexpected high values from US or MR elastography might indicate Fontan conduit failure (46, 109, 124). Conversely, a reduction in stiffness could indicate an improvement in Fontan circulation physiology or reduced congestion.

#### 6.3.5. Evaluation of liver nodules in FALD

Nodules of different sizes are frequent in FALD. They range from hyperplastic nodules to FNH, FNH-like lesions, regenerative nodules, malignant lesions including hepatocellular neoplasms (adenoma and carcinoma), and cholangiocarcinoma.

**Figure 10:**
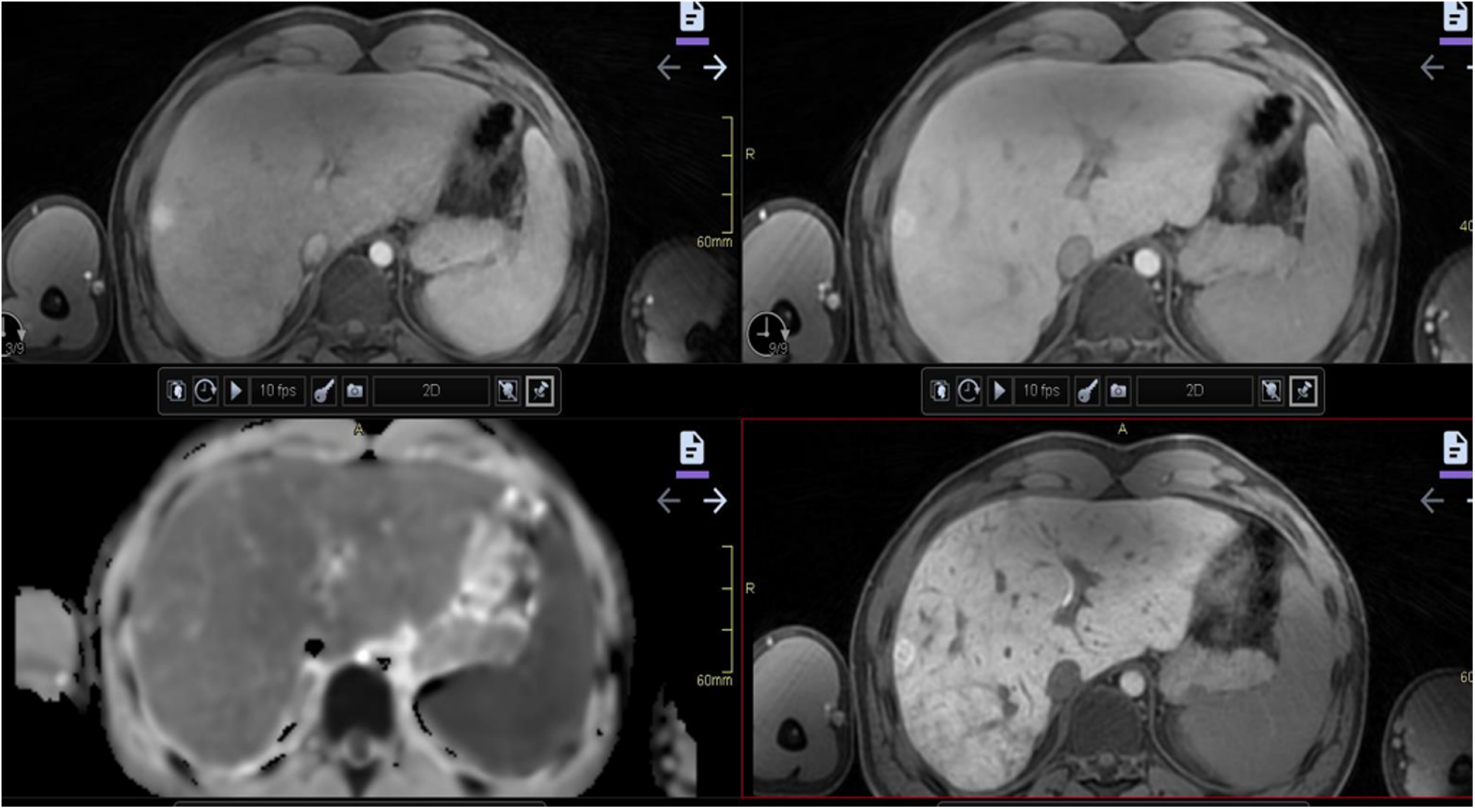
An 18-year-old boy born with double inlet left ventricle and pulmonary stenosis with a Fontan circulation. Satisfactory ventricular function was observed. Abdominal MRI was performed as a regular follow-up before transition to adult care. A 14 mm nodule not visible on ultrasound was found in the lateral part of the right liver lobe on MRI. Dynamic contrast-enhanced MRI with hepatocyte specific contrast agent was performed, revealing hyper-enhancement in arterial phase (upper left) and in late portal venous phase (upper right) as well as slightly irregular in hepatocyte phase (after 20 min) (lower right). The nodule is difficult to see clearly on the diffusion-weighted image (lower left). The nodule was interpreted with features of a probably benign nodule, and close follow-up with contrast-enhanced MRI was recommended. MRI, magnetic resonance imaging.

*Suspicious imaging findings of nodules that require further investigation with biopsy consist of (1) nodules >10 mm in diameter with rapid growth, (2) contrast enhancement pattern of early or late washout from CT or MRI, alternatively, hypo-or heterogeneous enhancement of the nodule on MRI hepatobiliary phase, and (3) An atypical signal on MRI basic sequences* (Table 2) (Figures 7, 10).

**Table 2:** Typical characteristics of benign and malign imaging features of nodules.

FALD nodules—appearance on CT/MR	Benign features	Malign features
Size/ growth	>10 mm stable size after 3–6 months	>10 mm, rapid growth (50% in <6 months or 100% in >6 months)
Form/contour	Well defined, regular, round	Ill defined, irregular shape/contour
MR T1 signal	Low signal	High signal
MR T2 signal	High signal	Low or isointense, heterogeneous
MR diffusion-weighted signal	Unrestricted	Restricted
MR/CT CE arterial phase	Hyper-enhancement	Hyper-enhancement
MR/CT CE portal venous phase	Hyper-enhancement or isointense to surrounding liver tissue	Hypo-enhancement to surrounding liver tissue^a^
MR hepatobiliary phase	NA	Hypo-enhancement, irregular enhancement

CE, Contrast enhancement; CT, computed tomography; MR, magnetic resonance.

^a^ Congestion with increased background signal in the liver can make nodules appear falsely hypo-enhancing in the portal venous phase.

**Figure 11:**
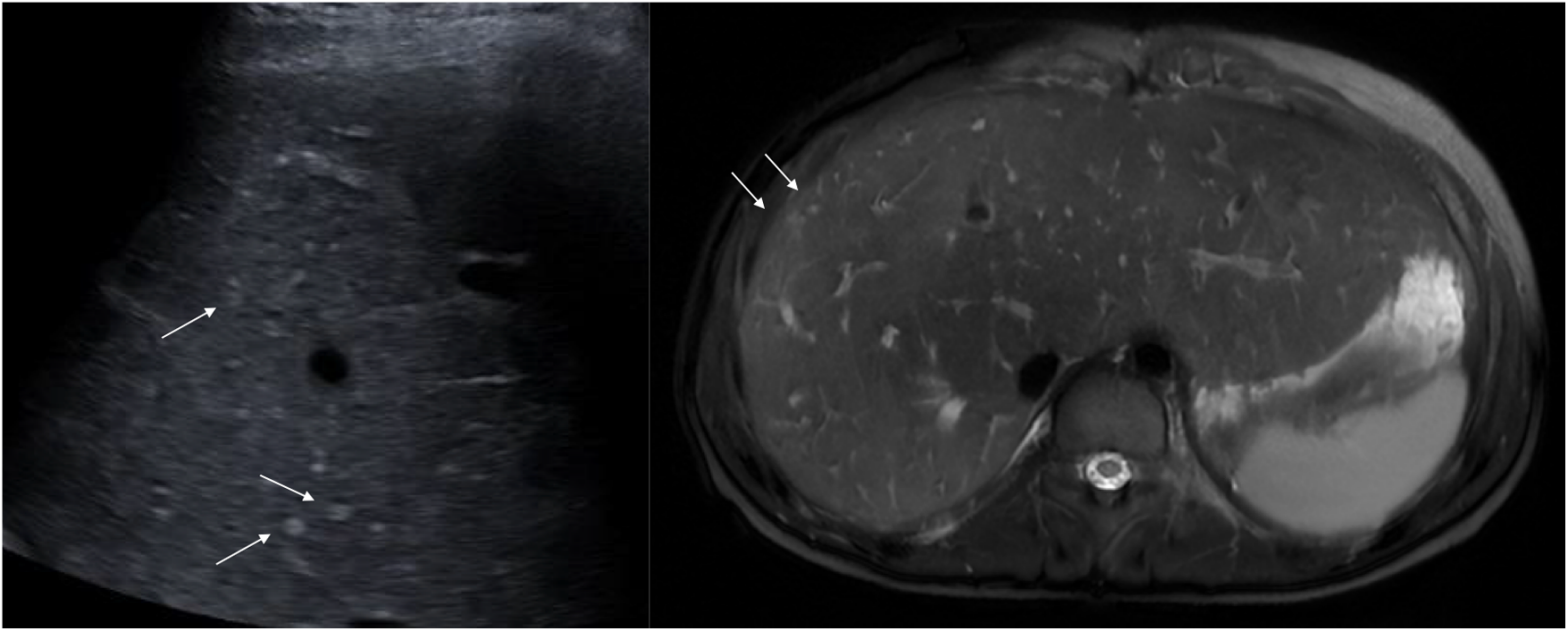
Typical hyperechogenic vascular nodules 3–5 mm (arrows) on ultrasound (left) and corresponding nodules with high signal on axial T2-weighted MRI (right). Note the generally increased T2 signal in the parenchyma as an expression of congestion (right). MRI, magnetic resonance imaging

With US, CT, and MRI, small nodular lesions are frequently observed predominantly in the right liver lobe (Figure 11) and in arterial contrast phase (99, 125). These hypervascular lesions are benign nodules and the most frequent finding in FALD livers, as well as in the pediatric population (74, 83, 86). Larger nodules may show overlapping features between large regenerative nodules and FNH, and are called FNH-like nodules. Larger lesions, which are >10 mm in diameter, may have different presentations owing to background congestion of the liver tissue. Diffusion-weighted imaging may show a diverse presentation, sometimes with restricted diffusion, for nodules with otherwise benign features (Table 2) (43, 96). These features may be in accordance with the Liver Imaging Reporting and Data System (LIRADS), which is frequently used to characterize and diagnose malignant liver lesions such as HCC (91). However, for the diagnosis of a malignant lesion in the setting of congestive hepatopathy, the LIRADS criteria are not reliable due to an increased risk of falsely diagnosing a malignancy (63, 91, 126–129).

Nevertheless, the LIRADS is still considered a useful tool for monitoring suspicion of HCC and guiding further evaluation, but it should be interpreted with caution (91, 126, 130). Consequently, all nodules with atypical imaging findings that suggest malignancy should undergo biopsy and be evaluated in referral centers (46, 57, 59, 63, 70).

#### 6.3.6. Surveillance of nodules

**Figure 12:**
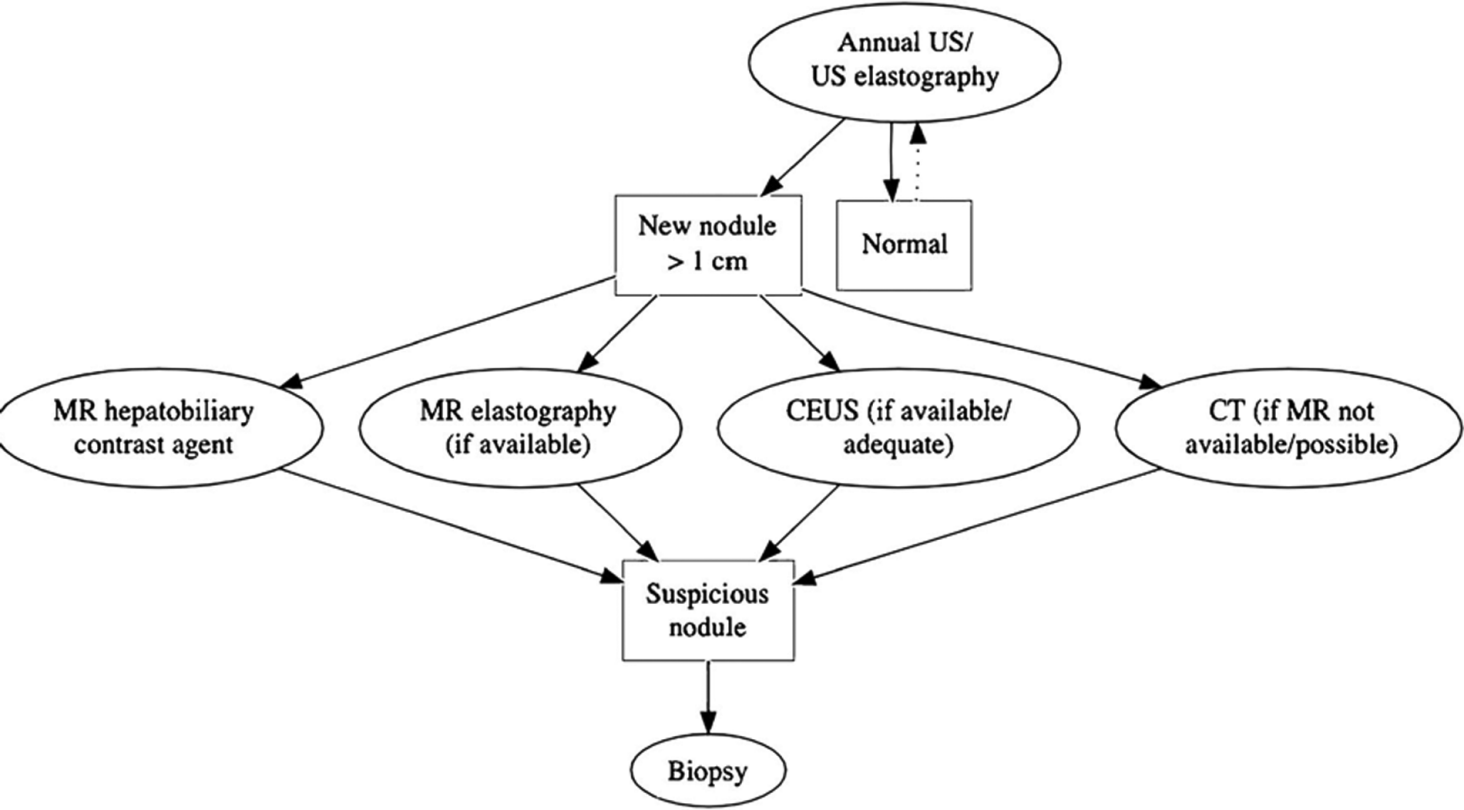
Flow chart of the proposed follow-up from the European Society of Pediatric Radiology, Abdominal Task Force. Reprint with permission from Perruca et al. Surveillance of Fontan associated liver disease: current standards and a proposal from the ESPR Abdominal Task Force. *Pediatr Radiol*. (2021) **51**:2598–2606.

Focal liver lesions >10 mm in diameter, irregular contours, capsules, or rapid growth (50% in less than 6 months or 100% in more than 6 months) should be evaluated with multiphase contrast-enhanced CT, or preferably MRI with a hepatocyte-specific contrast agent, for further and better characterization to assess for HCC and cholangiocarcinoma (Figures 7, 10, 12) (11, 56, 85, 131). Lesions presenting with a typical FNH-like appearance with homogeneous enhancement need to be followed up with CT or MRI within 6 months.

Even though a workup is in favor of an atypical but benign lesion, a follow-up scheme still needs to be followed in all cases to monitor for possible malignant transformation in the future (63, 91, 126).

## 7. Management—general considerations

The following recommendations are mainly for advanced stage disease:

*Pharmacological treatment:* Medication can be reviewed and adjusted to ameliorate the deleterious effects of the Fontan circulation. However, well-documented pharmacological treatment options are limited to agents that decrease pulmonary vascular resistance, such as sildenafil and bosentan (12, 132).

*Optimization of the Fontan circuit:* Determining any possible surgical or interventional method to improve hemodynamics in the Fontan circulation is important (27, 133). This includes interventions such as maintaining or enlarging fenestration, the stenting of pulmonary vascular obstructions, or other measures to ensure that there is no stenosis in the Fontan circuit and manage venovenous collaterals. There are no clear recommendations on when and how to intervene, and decisions are made on an individual case-by-case basis. In this regard, preprocedural imaging can provide information to select patients in need of treatment and further hemodynamic investigation, identifying stenosis in the anastomosis or development of collateral circulation. In particular, multimodal longitudinal follow-up for an individual patient is necessary to detect sudden hemodynamic and morphological changes in the Fontan circuit.

*Improvement of specific complications like PLE and PHTN:* PLE is diagnosed by elevated fecal *α*1-antitrypsin in combination with decreased serum albumin levels. Identifying lymphatic abnormalities and sites of intrahepatic obstructions and leakage can be important in evaluating possible interventional treatment strategies (100, 101). For specific cases, a few small studies from tertiary and specific centers have investigated dynamic intrahepatic MR lymphography to guide therapeutic and new catheter-based interventional treatments (103, 104).

Clinical signs of PHTN, such as ascites, variceal bleeding, and hepatic encephalopathy, occur in a more advanced stage, are associated with adverse outcomes in the Fontan circulation, and have implications for the decision to perform transplantation (49). The hepatic vein pressure gradient (HPVG) can be used to quantify PHTN in noncongestive cirrhosis and risk stratification of its complications. However, some studies have shown a poor correlation between the HPVG and FALD stage (76, 134). Evaluating serum albumin in ascites to measure gradient ascite total protein and serum BNP may help differentiate the origin of ascites as cardiac or hepatic (135). Nevertheless, cardiac failure, advanced liver disease, PHTN, and PLE may often overlap in patients with a Fontan circulation, which is important to consider when determining treatment.

### 7.1. Other liver-related management strategies

*Lifestyle:* Patients with a Fontan circulation should be specifically counseled regarding lifestyle measures to promote liver health, prevention, and the treatment (early or otherwise) of obesity. Increased weight has been associated with Fontan failure in adults (136). Such patients should be advised to engage in regular physical activity and a healthy diet, while avoiding alcohol, other hepatotoxic substances, and drugs.

*Infections:* Screening for hepatitis B and C is advised. To prevent an infection from the hepatitis A or B virus, both vaccines are generally recommended. In patients operated on before hepatitis C screening, which started in 1992, the prevalence of hepatitis C had a fivefold increase compared to the healthy population (137). Coincident diseases, such as non-alcoholic steatohepatitis, alcohol-induced liver disease, and other autoimmune liver diseases, can accelerate the development of FALD and should be identified and treated.

*Antithrombotic therapy:* Thromboembolic events are reported with an estimated prevalence of 17%–33% in patients with single-ventricle physiology and are associated with increased morbidity and mortality (138, 139). The risk for developing thrombosis is highest shortly after the Fontan circulation completed and at each previous stage of operation. Venous stasis is the most important predisposing factor, together with chronic low oxygen saturation and increased levels of hemoglobin and blood viscosity. Additionally, intrinsic factors contribute to fibrinolysis. Many authors support that anticoagulation is indicated in the presence or history of atrial thrombi, atrial arrhythmias, previous thromboembolic events, or a patent fenestration (140–142). On the other hand, there is still no consensus on the use of thromboprophylaxis for FALD and limited guidelines exist for pediatric patients (140, 142).

## 8. Management with heart or combined heart–liver transplantation

Studies have revealed a correlation between a high MELD XI score and decreased survival after transplantation (143, 144). Advanced liver cirrhosis and malignant transformation might prohibit heart transplantation or require combined liver and heart transplantation. The indication and patient selection for a combined liver–heart transplant remains a challenge in the absence of guidelines, with experience only from small outcome studies (83). The Organ Procurement and Transplantation Network database collects information on the outcomes of Fontan physiology and FALD from small single centers where differences or a lack of diagnostic codes are frequent, which make clear conclusions difficult (145). The indications for transplantation, therefore, differ between institutions, and for current practice regarding a cardiac vs. combined heart–liver transplant, the decision is case-specific. Liver transplantation alone is not recommended because the cause of liver failure and high CVP will remain and be deleterious for the new liver (65). A few studies have shown no differences in survival between cirrhotic and non-cirrhotic patients with Fontan circulation after heart transplantation (146–148). This indicates that severe fibrosis or cirrhosis, while compensated, is not a contraindication for isolated heart transplantation. Finally, the ability to reverse structural liver changes due to FALD after a heart transplant and conversion to biventricular circulation is not known (55), but in one study, regenerative capacity was reported to be higher than expected compared to those of other reversible liver diseases (133).

For adult patients with Fontan physiology, the number of heart and liver transplantations in the United States is steadily increasing (149). Many centers proceed with combined heart and liver transplantation because of the virtually universal presence of advanced liver fibrosis. Among pediatric patients, ventricular systolic dysfunction is the most common indication for cardiac transplantation; it is reported in 40%–60% of such patients (150–152). A few studies have revealed that in children, cardiac transplantation alone may be considered when indicated within a few years of the Fontan surgery (148, 153, 154). Initially, there were concerns of inferior survival in children transplanted for Fontan physiology compared to those transplanted for a non-CHD diagnosis (155). However, heart transplant outcomes for children in the current era have improved significantly, with better results than those of adult patients with a Fontan circulation. A pediatric heart transplant study described, in 2006, a 1-year survival rate of 76% after transplantation in children with a Fontan circulation, but a survival rate significantly lower than 91% in control participants without CHD (156, 157). In 2017, new data from an updated version of the above study reported a 1-year survival rate of 89% that was no longer distinguishable from the control group's survival rate, which was also confirmed in a more recent study (158, 159).

Combined heart–liver transplantation in pediatric patients is rare and data on outcomes are sparse. A single-center small series revealed limited but good early post-transplant outcomes in mostly older patients. In one study of nine patients with a median age of 20.7 years (range, 14.2–41.3 years), all with radiologic and pre-transplant evidence of cirrhosis, the 1-year survival rate without rejection was revealed to be 100% (160). Similar 1-year survival rates have been observed in other studies (161, 162). Recent studies indicate a decreased incidence of rejection after a combined heart–liver transplant, leading to the hypothesis that multiorgan transplantation induces immunotolerance and protection against rejection (162, 163); however, this needs to be further validated.

On the other hand, the development or regression of liver disease after heart transplantation in children has not been well studied. A few studies have used imaging in a follow-up of 1 year or longer after cardiac transplantation. In a retrospective study of 41 patients (median age, 12 years) with fibrosis, cirrhosis on pre-transplant imaging (29.2%) displayed less prevalence and regression in 19 patients’ imaging findings. There was no development of HCC or liver failure during short-term follow-up (164). However, continued post-transplant monitoring might be of importance, given the persistence of imaging findings suggestive of FALD, to some degree (164, 165).

## 9. Surveillance of FALD

The optimal surveillance strategy for FALD is still debated, especially in pediatric patients. Evidence-based studies are affected by short observational periods, and until now, the proposed published guidelines were mainly based on experience within adult age groups. Post-Fontan time is the most important predictor of advanced FALD. The risk of advanced disease is low up to 5 years after Fontan completion but increases significantly after 15 years (18, 40, 41, 54, 58). In a recent systematic review and retrospective study, HCC was unlikely within the first 10 years after the Fontan surgery (HCC was only diagnosed once before this time point), with an exponential risk markedly increasing in the third decade (59, 166). Hence, there is a general consensus that systematic monitoring for FALD and HCC is definitely indicated at 10 years post-Fontan and earlier if there are signs of Fontan failure or decreased liver function (11, 12, 56, 167). These investigations should include clinical examinations, blood tests, and imaging with US, CT, or MRI.

However, there is increasing evidence that FALD might develop earlier, within the first 5 years postoperatively, and that liver disease may even predate the Fontan procedure. Severe liver fibrosis in patients as young as 10 years of age has been described. The aim of surveillance is to diagnose FALD at a sufficiently early stage to consider interventions for optimizing the Fontan circulation in due time to prevent or delay the development of advanced fibrosis (167). There is growing evidence that an initial screening at baseline (before the Fontan creation) followed by two to three annual FALD screenings for children and adolescents, including clinical examination, biochemical evaluation, and liver Doppler US (with or without a liver stiffness assessment), might be favorable and allow early FALD detection. This scheme will provide the possibility for timely intervention in adolescents and young adults with failing Fontan physiology and might contribute to determining the optimal time point for transplantation. This is in line with the international guidelines from the American Heart Association of 2019 and the recommendations of the European Society of Pediatric Radiology (Figure 12) (11, 12, 27, 56, 167, 168).

As already mentioned, the role of liver biopsy in the follow-up of FALD also lacks consensus.

In screening young patients for liver disease and cancer development, psychological factors should also be considered. Lifelong surveillance for cancer from childhood may have important implications on the quality of life for patients and caregiver anxiety (169). The American Heart Association also recommends that such patients should be monitored in centers specialized in the Fontan circulation and with a multidisciplinary approach to diagnose and observe the different possible organ complications and development of FALD (12). This includes screening for malignancy, where imaging plays an important role, together with clinical scoring systems, biochemical workups, and biopsies.

## 10. Summary

FALD is a global liver disease that affects virtually all patients with a Fontan circulation, has a heterogeneous time course, and often commences in childhood. There are still many questions regarding the exact pathophysiology and development of FALD, and why certain patients develop malignancy. Lifelong follow-up and surveillance are mandatory to ensure the optimal timing for treatment and eventually transplantation. In this regard, monitoring with imaging plays an important role since clinical signs are indolent and serological markers are only slightly affected until the advanced stage of liver disease. Nevertheless, determining optimal surveillance protocols requires further investigation in longitudinal multicenter research studies. Importantly, Fontan physiology is a multiorgan disease where surveillance requires the involvement of a multidisciplinary team in cardiology, hepatology, anesthesiology, pathology, and radiology, in which all members are specialized in Fontan physiology and its complications.
